# Genetic Overlap Between Global Cortical Brain Structure, C-Reactive Protein, and White Blood Cell Counts

**DOI:** 10.1016/j.biopsych.2023.06.008

**Published:** 2023-06-20

**Authors:** Nadine Parker, Weiqiu Cheng, Guy F.L. Hindley, Kevin S. O’Connell, Sandeep Karthikeyan, Børge Holen, Alexey A. Shadrin, Zillur Rahman, Naz Karadag, Shahram Bahrami, Aihua Lin, Nils Eiel Steen, Thor Ueland, Pål Aukrust, Srdjan Djurovic, Anders M. Dale, Olav B. Smeland, Oleksandr Frei, Ole A. Andreassen

**Affiliations:** Norwegian Centre for Mental Disorders Research (NORMENT), Division of Mental Health and Addiction, Oslo University Hospital & Institute of Clinical Medicine, University of Oslo, Oslo, Norway; Norwegian Centre for Mental Disorders Research (NORMENT), Division of Mental Health and Addiction, Oslo University Hospital & Institute of Clinical Medicine, University of Oslo, Oslo, Norway; Norwegian Centre for Mental Disorders Research (NORMENT), Division of Mental Health and Addiction, Oslo University Hospital & Institute of Clinical Medicine, University of Oslo, Oslo, Norway; Psychosis Studies, Institute of Psychiatry, Psychology and Neurosciences, King’s College London, London, United Kingdom; Norwegian Centre for Mental Disorders Research (NORMENT), Division of Mental Health and Addiction, Oslo University Hospital & Institute of Clinical Medicine, University of Oslo, Oslo, Norway; Norwegian Centre for Mental Disorders Research (NORMENT), Division of Mental Health and Addiction, Oslo University Hospital & Institute of Clinical Medicine, University of Oslo, Oslo, Norway; Norwegian Centre for Mental Disorders Research (NORMENT), Division of Mental Health and Addiction, Oslo University Hospital & Institute of Clinical Medicine, University of Oslo, Oslo, Norway; Norwegian Centre for Mental Disorders Research (NORMENT), Division of Mental Health and Addiction, Oslo University Hospital & Institute of Clinical Medicine, University of Oslo, Oslo, Norway; Norwegian Centre for Mental Disorders Research (NORMENT), Division of Mental Health and Addiction, Oslo University Hospital & Institute of Clinical Medicine, University of Oslo, Oslo, Norway; Norwegian Centre for Mental Disorders Research (NORMENT), Division of Mental Health and Addiction, Oslo University Hospital & Institute of Clinical Medicine, University of Oslo, Oslo, Norway; Norwegian Centre for Mental Disorders Research (NORMENT), Division of Mental Health and Addiction, Oslo University Hospital & Institute of Clinical Medicine, University of Oslo, Oslo, Norway; Norwegian Centre for Mental Disorders Research (NORMENT), Division of Mental Health and Addiction, Oslo University Hospital & Institute of Clinical Medicine, University of Oslo, Oslo, Norway; Norwegian Centre for Mental Disorders Research (NORMENT), Division of Mental Health and Addiction, Oslo University Hospital & Institute of Clinical Medicine, University of Oslo, Oslo, Norway; Institute of Clinical Medicine, University of Oslo, Oslo, Norway; Research Institute of Internal Medicine, Oslo University Hospital, Oslo, Norway; KG Jebsen Thrombosis Research and Expertise Centre, University of Tromsø, Tromsø, Norway; Institute of Clinical Medicine, University of Oslo, Oslo, Norway; Research Institute of Internal Medicine, Oslo University Hospital Rikshospitalet, Oslo, Norway; Section of Clinical Immunology and Infectious Disease, Oslo University Hospital Rikshospitalet, Oslo, Norway; Department of Medical Genetics, Oslo University Hospital, Oslo, Norway; Norwegian Centre for Mental Disorders Research, Department of Clinical Science, University of Bergen, Bergen, Norway; Center for Multimodal Imaging and Genetics, University of California San Diego, La Jolla, California; Department of Psychiatry, University of California, San Diego, La Jolla, California; Department of Neurosciences, University of California San Diego, La Jolla, California; Department of Radiology, University of California San Diego, La Jolla, California; Norwegian Centre for Mental Disorders Research (NORMENT), Division of Mental Health and Addiction, Oslo University Hospital & Institute of Clinical Medicine, University of Oslo, Oslo, Norway; Norwegian Centre for Mental Disorders Research (NORMENT), Division of Mental Health and Addiction, Oslo University Hospital & Institute of Clinical Medicine, University of Oslo, Oslo, Norway; Norwegian Centre for Mental Disorders Research (NORMENT), Division of Mental Health and Addiction, Oslo University Hospital & Institute of Clinical Medicine, University of Oslo, Oslo, Norway

## Abstract

**BACKGROUND:**

For many brain disorders, a subset of patients jointly exhibit alterations in cortical brain structure and elevated levels of circulating immune markers. This may be driven in part by shared genetic architecture. Therefore, we investigated the phenotypic and genetic associations linking global cortical surface area and thickness with blood immune markers (i.e., white blood cell counts and plasma C-reactive protein levels).

**METHODS:**

Linear regression was used to assess phenotypic associations in 30,823 UK Biobank participants. Genome-wide and local genetic correlations were assessed using linkage disequilibrium score regression and local analysis of covariance annotation. The number of shared trait-influencing genetic variants was estimated using MiXeR. Shared genetic architecture was assessed using a conjunctional false discovery rate framework, and mapped genes were included in gene-set enrichment analyses.

**RESULTS:**

Cortical structure and blood immune markers exhibited predominantly inverse phenotypic associations. There were modest genome-wide genetic correlations, the strongest of which were for C-reactive protein levels (*r*_g_surface_area_ = −0.13, false discovery rate–corrected *p* = 4.17 × 10^−3^; *r*_g_thickness_ = −0.13, false discovery rate–corrected *p* = 4.00 × 10^−2^). Meanwhile, local genetic correlations showed a mosaic of positive and negative associations. White blood cells shared on average 46.24% and 38.64% of trait-influencing genetic variants with surface area and thickness, respectively. Additionally, surface area shared 55 unique loci with the blood immune markers while thickness shared 15. Overall, monocyte count exhibited the largest genetic overlap with cortical brain structure. A series of gene enrichment analyses implicated neuronal-, astrocytic-, and schizophrenia-associated genes.

**CONCLUSIONS:**

The findings indicate shared genetic underpinnings for cortical brain structure and blood immune markers, with implications for neurodevelopment and understanding the etiology of brain-related disorders.

Classically, the brain was considered an immune-privileged organ, but it is now clear that there is substantial immune-associated activity within the brain and crosstalk between central (i.e., brain) and peripheral (i.e., blood) immune signaling. Mediators of the immune system, such as various cytokines, have been found to play a role in brain development and maintenance of synaptic plasticity (1). Moreover, peripheral-central immune crosstalk occurs through inflammatory-induced blood-brain barrier disruption, circumventricular organs, and vagal nerve transmission (2,3). This crosstalk plays a hypothesized role in many brain-related disorders such as neurodevelopmental (4), psychiatric (5–8), and neurodegenerative (9–11) disorders. For example, a study of outpatients with major depressive disorder reported a strong correlation (*r* = 0.86) between plasma and cerebrospinal fluid levels of C-reactive protein (CRP), an acute-phase biomarker of inflammation (12). Moreover, studies have shown that peripheral immune cells may infiltrate brain tissue, exaggerating features of neurodegenerative and psychiatric pathology (11,13).

Notably, brain structure serves as a potential endophenotype for the many brain-related disorders (14,15). For example, findings from the ENIGMA (Enhancing Neuro Imaging Genetics through Meta Analysis) Consortium include widespread reductions in both cortical surface area and thickness in patients with schizophrenia, while patients with bipolar disorder exhibit reductions in thickness but less so in surface area (16). A shared genetic architecture between these two cortical features and peripheral immune markers may contribute to the pathophysiology of brain disorders.

White blood cell (WBC) counts and CRP are among the most clinically relevant peripheral biomarkers of inflammation and immune activation. However, WBC counts and CRP are nonspecific immune markers that are more representative of broad domains of immune activity. WBCs play a role in innate and adaptive immunity while secreting an array of factors including both pro- and anti-inflammatory cytokines as well as neurotrophic factors (17,18). Moreover, the brain’s resident immune cells, microglia, have similar yolk sac origins as the first wave of WBCs generated during embryonic development (19,20). Similarities in factor secretion and immune cell origins in brain and blood provide potential biological mechanisms linking the brain and blood immune markers.

Both WBCs and CRP are associated with alterations in brain structure. WBC count has been linked to reductions in total brain volume as well as gray matter volume (21). Patients with schizophrenia and depression have been reported to exhibit an inverse relationship between blood levels of CRP and thickness of the cerebral cortex (22–24). Also, several studies of healthy middle-aged and older participants have found reduced gray matter volumes in association with elevated levels of CRP (25–28). Here, we focus on cortical surface area and thickness, two subcomponents of cortical volume with distinct genetic architectures (29,30).

Recent large-scale genetic studies of cortical brain structure, CRP, and WBC counts provide resources to study the genetic interplay between these traits (30–32). There is little known about the shared genetic architecture even given evidence for a link to neurodevelopment, central-peripheral immune crosstalk, the potential psychiatric-brain immune link, and previous phenotypic associations. Therefore, we proposed to investigate the genetic overlap between global cortical brain structure (i.e., surface area and thickness) and blood immune markers (i.e., WBC counts and CRP). Here, we begin with a brief phenotypic analysis. Then, we use unique tools that go beyond global genetic correlations to estimate 1) local genetic correlations, 2) the number of shared genetic variants, 3) specific shared genetic loci, and 4) gene enrichment for biological mechanisms and disorders. The results of this study may elucidate pleiotropic effects in the brain and immune system, with implications for brain development and brain-related disorders.

## METHODS AND MATERIALS

### Phenotypic Analysis

Data were acquired from the UK Biobank (application No. 27412) to assess the association between cortical brain structure and the blood immune markers. Measures of global surface area and cortical thickness were provided for 40,055 participants. For WBC counts (basophil, eosinophil, lymphocyte, monocyte, neutrophil, and leukocyte [i.e., total WBC] count), we acquired measures based on blood sampling 1) at the time of recruitment (*n* = 478,168) and 2) at the time of magnetic resonance imaging (MRI) scan (*n* = 5865). Plasma CRP levels were measured only for recruitment samples. Details on the processing of MRI scans and blood samples are in the Supplemental Methods in Supplement 1.

The associations between cortical brain structure (i.e., surface area and thickness) and each blood immune marker (i.e., CRP and WBC counts) were assessed using linear regression in R (version 4.0.0). Participants with a history of stroke, dementia, or psychiatric diagnoses were excluded. Blood immune markers were log transformed with the exception of basophil and eosinophil count, which were square root transformed. Next, all cortical and immune metrics were standardized to *z* scores. Outliers >4 standard deviations from the mean were removed. Age, age^2^, sex, body mass index, and MRI site were included as covariates. For models using blood markers measured from recruitment samples, the difference in age from baseline to MRI scan was added as an additional covariate. Sensitivity analyses were conducted for the inclusion of blood pressure and smoking status as covariates (Supplemental Methods in Supplement 1).

### Genetic Data

Genome-wide association study (GWAS) summary statistics were acquired for global cortical surface area and thickness from the ENIGMA Consortium (*n* = 33,992) (30). To avoid sample overlap for the conjunctional false discovery rate (conjFDR) analyses (see below), summary statistics excluding the UK Biobank cohort were also acquired. For validation of discovered shared variants, a GWAS from the UK Biobank (*n* = 33 735) was utilized (33). GWAS summary statistics for CRP were acquired from the Neale Lab using 361,194 UK Biobank participants (http://www.nealelab.is/blog/2019/9/16/biomarkers-gwas-results). For validation of discovered shared variants, a GWAS by the CHARGE (Cohorts for Heart and Aging Research in Genomic Epidemiology) Consortium (*n* = 204,402) was utilized (31). The Blood Cell Consortium (http://www.mhi-humangenetics.org/en/resources/) provided GWAS summary statistics for all WBC counts (*n* = 563,085) (32). To avoid sample overlap for conjFDR analyses, we used summary statistics derived from the UK Biobank sample only. For validation of discovered shared variants, summary statistics from a sample of 151,807 East Asian participants was utilized (34). This sample did not include summary statistics for leukocyte count. A list of the GWASs utilized is provided in Table S1 in Supplement 2.

### Genetic Correlations

Pairwise genetic correlations at the global (i.e., genome-wide) and local (i.e., within a region of the genome) levels were quantified using linkage disequilibrium score regression (LDSR) (35) and local analysis of covariant annotation (LAVA) (36), respectively (Supplemental Methods in Supplement 1). Local analysis of covariant annotation estimates local genetic correlations across 2495 genome-wide regions. To adjust for multiple comparisons, the Benjamini-Hochberg method was applied across all pairwise comparisons.

### Quantifying Overlapping Variants

To estimate the number of overlapping variants between pairs of traits, we used bivariate MiXeR version 1.3 (https://github.com/precimed/mixer) (37,38). MiXeR uses a Gaussian causal mixture model to estimate the number of common variants with a direct effect on the trait of interest excluding effects due to linkage disequilibrium; these variants are referred to as “trait-influencing variants” (Supplemental Methods in Supplement 1). Given 2 traits of interest, MiXeR models the number of trait-influencing variants that are unique to each trait (nonoverlapping) as well as the number of shared trait-influencing variants (overlapping).

### ConjFDR to Identify Shared Loci

For a pair of traits, conditional quantile-quantile plots are constructed. One trait is selected as the primary trait, and quantile-quantile plots are generated for several *p* value thresholds (*p* = 1, .1, .01, .001) in the secondary trait. A leftward deflection away from the null (diagonal) with a decrease in *p*-value threshold is indicative of strong enrichment of the primary trait conditioned on the secondary. Cross-trait enrichment for both traits conditioned on each other is a requirement for a valid conjFDR analysis (https://github.com/precimed/pleiofdr) (39).

Enrichment observed in conditional quantile-quantile plots are used to compute a conditional FDR (condFDR) for each single nucleotide polymorphism (SNP) (Supplemental Methods in Supplement 1). The condFDR is a conservative estimate of observed *p* values/expected *q* values and is defined as the posterior probability that a SNP is null for the primary phenotype given that the *p* values for associations with both phenotypes are as small as or smaller than their observed *p* values. For a pair of traits, 2 sets of condFDR values are computed such that each trait plays the role of primary and secondary trait. The conjFDR is defined as the maximum of the 2 condFDR values for each SNP. The conjFDR is considered the posterior probability that a SNP is null for one or both traits given that the *p* values for associations with both traits are as small as or smaller than their observed *p* values. A significance threshold of conjFDR < 0.05 was applied for all analyses. These analyses were conducted with the exclusion of the major histocompatibility complex and 8p23 inversion genomic regions. All conjFDR analyses were conducted using nonoverlapping GWAS samples.

### Gene Mapping and Quantifying Overlapping Loci

The functional mapping and annotation procedure (https://fuma.ctglab.nl/) was used to identify independent loci and map those loci to genes (Supplemental Methods in Supplement 1). In brief, to annotate conjFDR-identified loci, positional, expression quantitative trait loci, and chromatin interaction gene mapping were considered. Genes that were identified by at least 2 of the 3 mapping procedures were used for enrichment analyses.

Overlapping loci shared between cortical brain structures and each of the immune markers were identified using the “foverlaps” function in R package “data.table.” To determine the pattern of effect directions across shared loci, the proportion of lead SNPs with discordant effect directions was calculated for each cortical brain structure and immune trait pairing.

### Validation of Lead SNP Effect Directions

Using an independent GWAS sample, validation of lead SNPs was conducted with an exact binomial test to determine whether the proportion of lead SNPs with concordant signs in the original and replication sample was significantly above 50%.

### Enrichment Analyses

The main set of enrichment analyses were conducted for a combined set of genes mapped to all shared loci between a cortical measure and all associated immune markers (i.e., surface area–immune genes and thickness-immune genes). Follow-up analyses were conducted separately for each individual trait pairing. Functional mapping and annotation GENE2FUNC was used to determine enrichment for gene ontology groups. Cell type and disorder gene enrichment were conducted with R software using hypergeometric tests. Corrections for multiple comparisons were done using the Benjamini-Hochberg method. Cell type–specific gene sets were acquired from 3 human brain resources and 1 mouse brain resource (40–43). Curated lists of disorder-related genes were acquired from DisGeNET using the R package “disgenet2r.” Additional details are provided in Supplemental Methods in Supplement 1.

## RESULTS

### Phenotypic Associations

**Figure 1.**
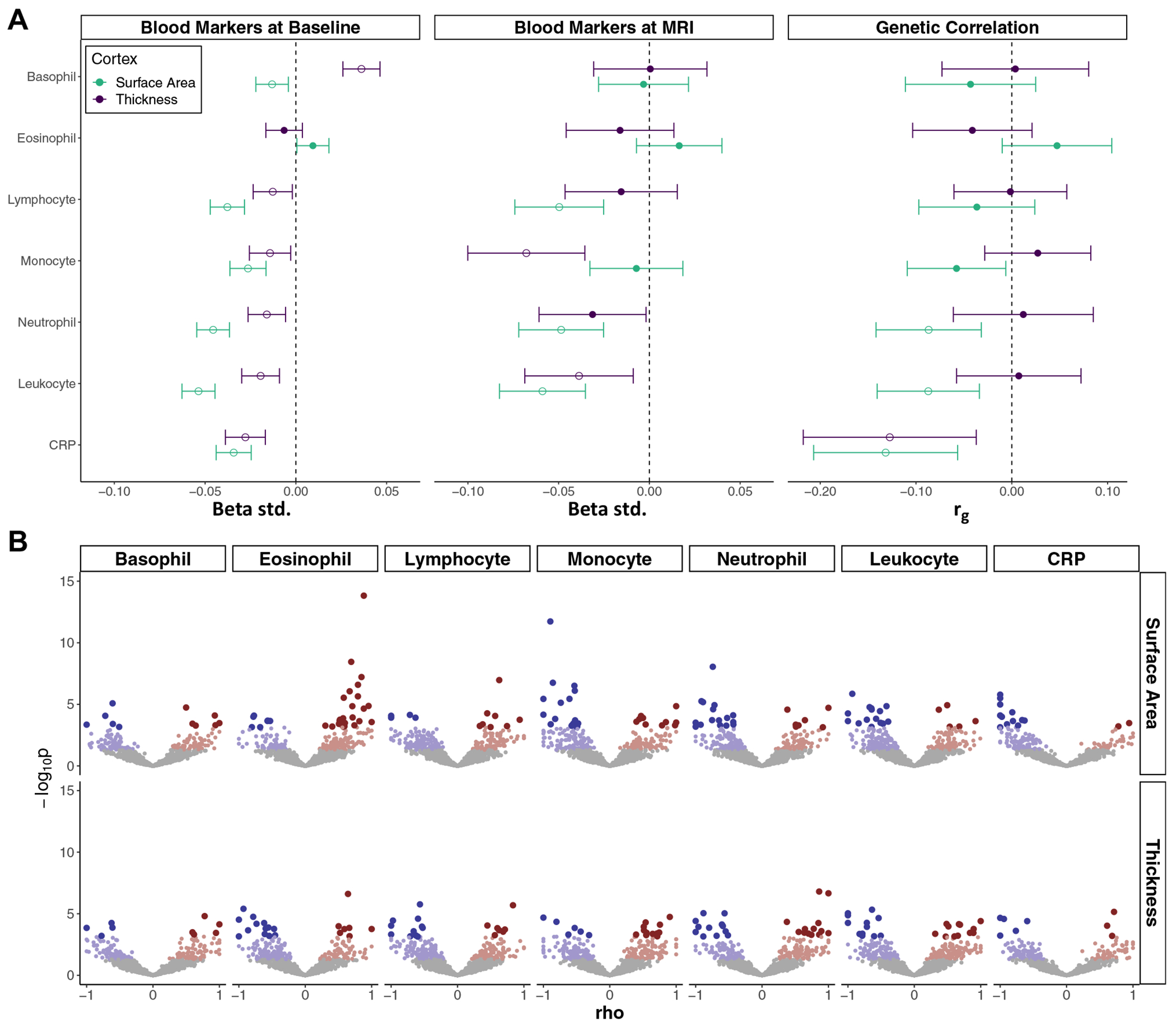
Phenotypic associations and genetic correlations. **(A)** A plot displaying the phenotypic association and global (genome-wide) genetic correlations between cortical brain structure and the blood immune markers. Far left: the phenotypic association, using linear regression, between blood immune markers, measured at the baseline recruitment time point, and cortical brain structure. Center: the phenotypic association, using linear regression, between the blood immune markers, measured at the time of MRI visit, and cortical brain structure. Far right: the global genetic correlation, using linkage disequilibrium score regression, between cortical brain structure and the blood immune markers. Open circles represent significant associations after false discovery rate correction for multiple comparisons. **(B)** A volcano plot displaying the local genetic correlations, using local analysis of covariance annotation, between cortical brain structure and the blood immune markers. Gray points are nonsignificant correlations. Blue points represent negative correlations, and red points (positive) represent correlations with nominal significance (*p* < .05). Darker and larger red/blue points represent correlations that surpassed false discovery rate correction. CRP, C-reactive protein; MRI, magnetic resonance imaging.

Using data from 37,312 UK Biobank participants, the associations between cortical brain structure and the blood immune markers sampled at recruitment were assessed. The mean age of participants was 63.55 (SD = 7.53) years, and the sample was 52.9% female. On average, there were 8.78 (SD = 1.77) years between recruitment and MRI scans. Surface area was negatively associated with all immune markers except eosinophil count (Figure 1A). Cortical thickness exhibited a negative association with all immune markers except for eosinophil (β_std_ = −6.46 × 10^−3^, *p* = .26) and basophil (β_std_ = 3.61 × 10^−2^, *p* = 2.34 × 10^−11^) counts.

For 5548 participants with a mean age of 61.88 (SD = 7.53) (51.7% female), immune markers measured at the time of imaging were used. Trends in association were similar to those reported for the above baseline analysis (*r* = 0.69, *p* = .01), and WBC counts were subtly higher than baseline (Table S2 in Supplement 2). With a smaller sample size and larger confidence intervals, there were fewer significant associations (Figure 1A). Sensitivity analyses suggest that the addition of smoking and blood pressure as covariates may subtly reduce associations for most pairs of phenotypes but may only result in the loss of association for 1) thickness and neutrophil count and 2) thickness and leukocyte count (Figure S1 in Supplement 1).

### Genetic Correlations

There were negative genome-wide correlations between cortical surface area and CRP (*r*_g_ = −0.13, *p*_FDR_ = 4.17 × 10^−3^), monocyte (*r*_g_ = −0.06, *p*_FDR_ = 4.87 × 10^−2^), neutrophil (*r*_g_ = −0.09, *p*_FDR_ = 4.56 × 10^−3^), and leukocyte (*r*_g_ = −0.09, *p*_FDR_ = 4.56 × 10^−3^) counts (Figure 1A). For cortical thickness, a negative genetic correlation was observed with CRP (*r*_g_ = −0.13, *p*_FDR_ = 4.00 × 10^−2^) only. Across all phenotypes, genetic correlations ranged widely in magnitude (*r*_g_ = −0.32 to 0.91) (Figure S2 in Supplement 1).

The general pattern of local genetic correlations shows a shift toward more negative correlations, coinciding with genome-wide correlations (Figure 1B). However, a complex mosaic of negative and positive local genetic correlations is visible. The number of significant local genetic correlations for each pairwise association is provided in Table S3 in Supplement 2.

### Genetic Overlap Beyond Genetic Correlation

**Figure 2.**
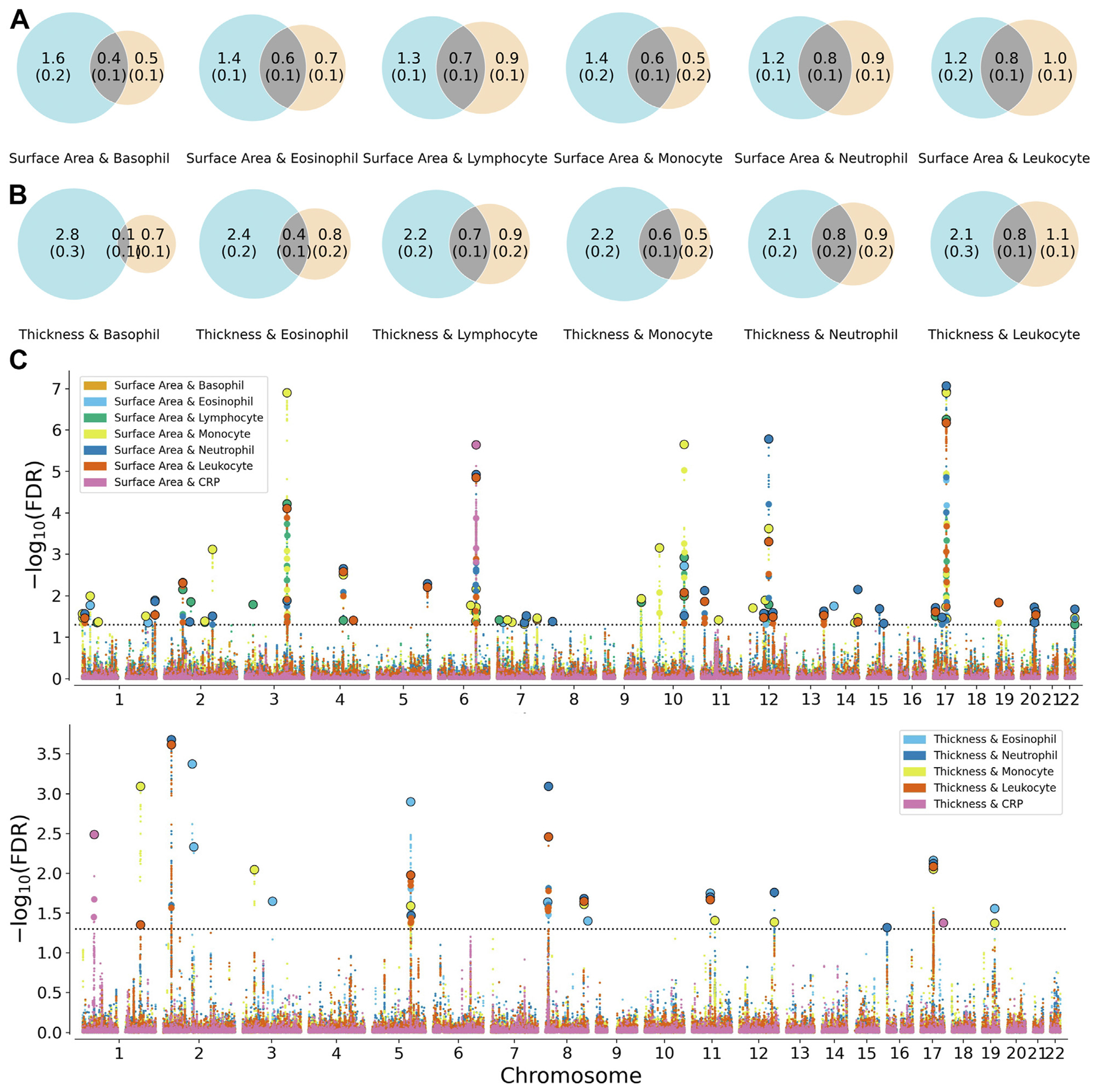
Bivariate MiXeR and conjunctional FDR results. **(A)** Venn diagrams representing the genetic overlap (gray) between surface area (blue) and the white blood cell counts (orange). **(B)** Venn diagrams representing the genetic overlap (gray) between thickness (blue) and the white blood cell counts (orange). For plots A and B, the size of the circle depicts the polygenicity of the phenotype. Numeric values represent the estimated number of trait-influencing variants, with the standard deviations of estimates in brackets. These values are in thousands rounded to the nearest 10th (e.g., 1.5 = 1500 variants). **(C)** Manhattan plot displaying conjunctional loci associated with immune markers and surface area (top) as well as thickness (bottom). Larger circles outlined in black represent lead variants. CRP, C-reactive protein; FDR, false discovery rate.

MiXeR was used to determine the number of overlapping variants between trait pairs (Table S4 in Supplement 2; Figure S3 in Supplement 1). Overall, WBC counts were less polygenic than cortical surface area and thickness (Figure 2). WBC counts shared, on average, 46.24% of their variants with cortical surface area. The largest overlap was with monocyte count (50.55%) and the lowest was with lymphocyte count (43.94%). Overall, WBC counts shared, on average, 38.64% of variants with cortical thickness. Again, the largest overlap was with monocyte count (55.60%), while the lowest was with basophil count (11.72%). CRP results were not included due to inadequate model fit, which was presumably due to low polygenicity (Figure S3 in Supplement 1) (37).

To identify shared genetic loci, conjFDR analyses were performed. Cross-trait SNP enrichment for cortical surface area was observed for all immune markers (Figure S4 in Supplement 1). Genetic variants jointly associated with surface area and immune markers were distributed across the genome (Figure 2C; Table S5 in Supplement 2), with 55 unique loci identified (Table S6 in Supplement 2), and monocyte count had the greatest quantity of shared loci (*n* = 34). Shared loci with surface area overlapped across the 7 blood immune markers (Figure S6 in Supplement 1). In particular, a locus on chromosome 10 was shared between surface area and all WBC counts but not CRP. The single locus shared between surface area and CRP on chromosome 6 overlapped with loci found with monocyte, neutrophil, and leukocyte counts. Consistent with the local genetic correlations, most lead SNPs were discordant between surface area and each of the immune markers except eosinophil count, which had a concordant effect for 80% of the lead SNPs (Table S7 in Supplement 2).

Cross-trait SNP enrichment was observed for cortical thickness across CRP, eosinophil, monocyte, neutrophil, and leukocyte counts (Figure S5 in Supplement 1), where a total of 15 unique shared loci were identified (Table S6 in Supplement 2). Contributing most to the shared genetic architecture, eosinophils, monocytes, and neutrophils each shared 6 loci with cortical thickness. There was limited overlap in shared loci across each of the pairwise comparisons (Figure S6 in Supplement 1). A locus on chromosome 5 showed overlap between thickness and eosinophil, monocyte, neutrophil, and leukocyte counts but not CRP. The 2 thickness and CRP loci did not overlap with loci shared between thickness and any WBC count. All lead SNPs exhibited a greater proportion of discordant effects between thickness and the immune markers (Table S7 in Supplement 2).

### Lead SNP Validation in Independent Samples

Cortical surface area exhibited concordant effect directions for 96.4% of lead SNPs shared across the blood immune markers in an independent sample. For thickness, 88.9% of lead SNPs exhibited concordant effects in an independent sample. Lead SNPs shared between WBC counts and surface area had concordant effect directions ranging from 50% to 78.1% in independent samples. However, none of the 3 lead SNPs for basophil count replicated effect direction. For WBC counts and cortical thickness, 50% to 100% of lead SNPs had concordant effect directions in independent samples. CRP had consistent effect directions in 100% and 50% of lead SNPs for surface area and thickness, respectively. Details are provided in Table S8 in Supplement 2.

### Functional Enrichment

**Figure 3.**
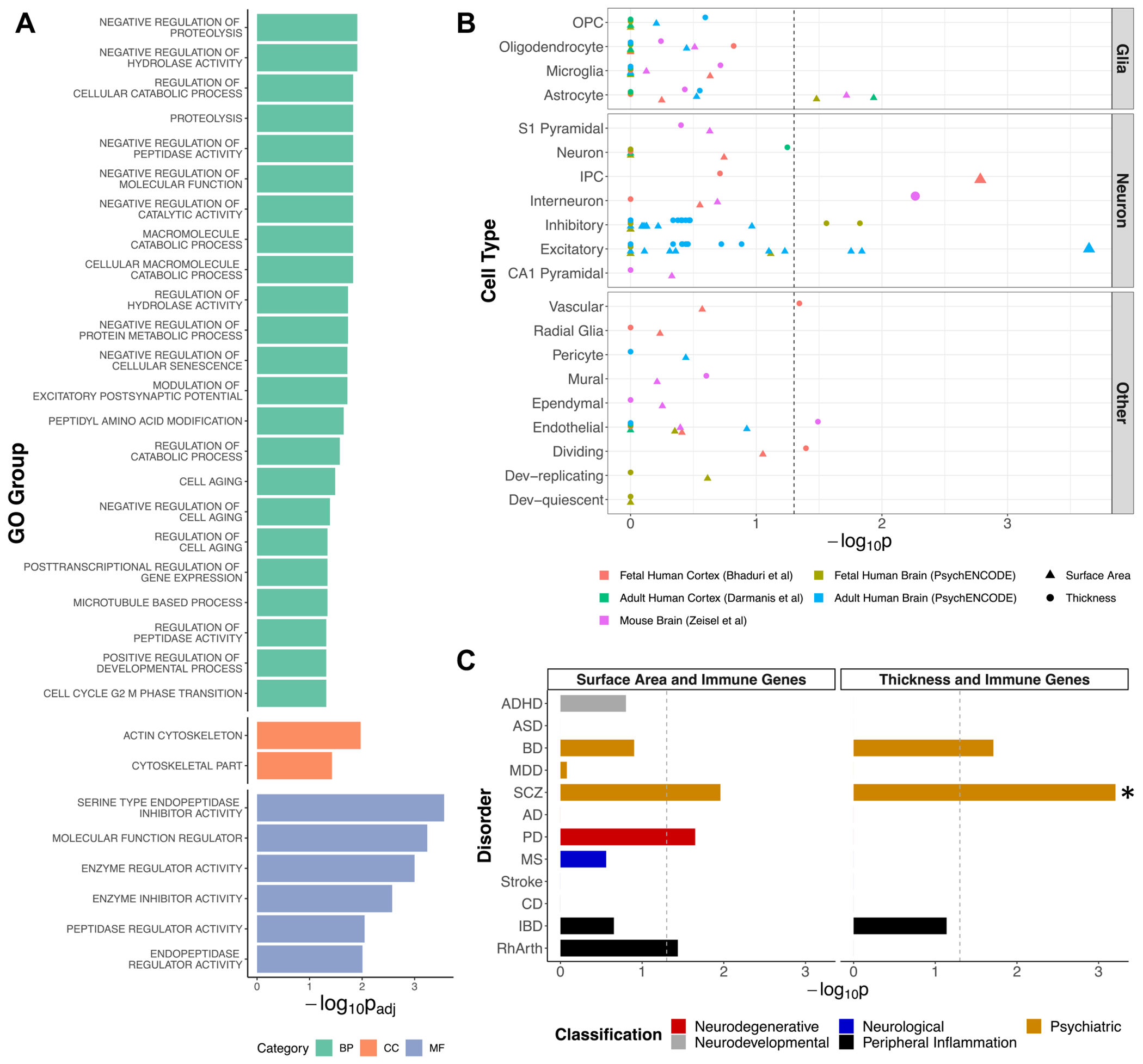
Enrichment analyses for genes mapped to conjunctional false discovery rate loci. **(A)** Gene ontology group enrichment for surface area–immune genes. All groups depicted survived correction for multiple comparisons. Each category (BP, CC, and MF) is displayed as a different color. **(B)** Cell type–specific enrichment for genes mapped to conjunctional loci shared by surface area and all 7 immune markers (triangles) as well as thickness and the 5 associated immune markers (circles). The 3 larger points represent associations that survived correction for multiple comparisons. **(C)** Enrichment analyses for disorder-associated genes among genes mapped for conjunctional loci for immune and surface area (left) as well as thickness (right). The asterisk signifies significance after correction for multiple comparisons. The vertical dotted lines in panels **(B)** and **(C)** represent *p* = .05 (nominal significance). AD, Alzheimer disease; ADHD, attention-deficit/hyperactivity disorder; ASD, autism spectrum disorder; BD, bipolar disorder; BP, biological process; CC, cellular component; CD, Crohn’s disease; Dev, developmental; GO, gene ontology; IBD, irritable bowel disease; IPC, intermediate progenitor cell; MDD, major depressive disorder; MF, molecular function; MS, multiple sclerosis; OPC, oligodendrocyte progenitor cell; PD, Parkinson’s disease; RhArth, rheumatoid arthritis; SCZ, schizophrenia.

Surface area–immune genes (*n* = 409) (Table S9 in Supplement 2) showed enrichment for several gene ontology groups involved in catabolic activity, modulation of excitatory postsynaptic potentials, regulation of developmental processes and cell aging/death, and cytoskeletal and enzymatic function (Figure 3A; Table S10 in Supplement 2). Thickness-immune genes (*n* = 113) (Table S9 in Supplement 2) were not enriched for any gene ontology group.

Surface area–immune genes were nominally enriched for 3 sources of astrocyte genes (Figure 3B), and *SLC30A10* was among the overlapping genes. From the 2 neuronal enriched cell types, the *KLC1* and *SYT1* genes overlapped. Enrichment for neuronal and nominal enrichment for vascular cell–specific genes was observed among the thickness-immune genes (Figure 3B). The gene *DPYSL5*, among the interneuron enriched genes, also overlapped with inhibitory neuron genes, which showed nominal enrichment (Table S13 in Supplement 2).

Surface area–immune genes were nominally enriched for schizophrenia, Parkinson’s disease, and rheumatoid arthritis genes (Figure 3C). Meanwhile, thickness-immune genes were enriched for schizophrenia genes, and there was nominal enrichment for bipolar disorder genes.

The results of all pairwise analyses are reported in Table S11 in Supplement 2 for gene ontology enrichment, Table S12 in Supplement 2 for cell-type enrichment for surface area and immune markers, Table S13 in Supplement 2 for cell-type enrichment for thickness and immune markers, and Table S14 in Supplement 2 for disorder gene enrichment.

## DISCUSSION

Leveraging large-scale data and various techniques that capture genetic overlap, this study uncovered both phenotypic and genetic relationships between global cortical brain structure and blood immune markers. Modest genome-wide genetic correlations were observed, yet local genetic correlations showed a mosaic of positive and negative associations that likely reflect the complex relationships between these brain and blood traits. WBCs shared on average 46.24% and 38.64% of their genetic variants with surface area and thickness, respectively. Additionally, surface area shared 55 unique loci with the immune markers while thickness shared 15. Shared genetics between cortical brain structure and the immune markers implicated potential underlying mechanisms and several disorders, in particular schizophrenia. Overall, cortical surface area had stronger phenotypic and genetic associations with the blood immune markers than thickness. Among the immune markers, monocyte count exhibited the largest amount of genetic overlap with each cortical feature, suggesting a role for innate immunity and inflammation. These findings provide evidence for a shared genetic component between cortical brain structure and clinically relevant peripheral immune markers, with implications for brain disorders.

Cortical brain structure and the blood immune markers exhibited modest negative genome-wide genetic correlations. A similar pattern of genetic correlations between cortical thickness, CRP, and WBC counts was found in a study of 50 blood biochemical traits and cortical brain structure (44). We further investigated the shared genetic architecture to find a mosaic of mixed effect directions. These mixed effects may underly the complex role of cytokines in brain and blood as well as their potential crosstalk. Depending on the cytokine, the timing, location, and length of exposure, as well as the presence of other cytokines, may have advantageous or deleterious effects on brain tissue, blood-brain barrier integrity, or immune activity (1,45,46). Various cytokines play important roles in brain development and postdevelopmental maintenance of synaptic activity (1), which is consistent with the observed positive associations between cortical structure and blood immune markers. Additionally, evidence that WBCs, such as basophils and eosinophils, produce and respond to neurotrophic factors also helps to explain the presence of positive phenotypic and genetic associations (18,47). Additional genetic investigation in this area may help researchers disentangle this complex relationship.

In the phenotypic analyses, a subtle inverse association was observed between cortical brain structure and plasma CRP levels as well as most of the WBC counts. This is consistent with several previous studies in which reductions in cortical and total gray matter volumes with elevated CRP levels (25,27) and WBC counts were reported (7,21). Our results provide evidence that volumetric associations in the cortex may be driven by reductions in surface area and to a lesser extent cortical thickness. Notably, a positive association was observed for basophils with thickness, but this was not supported by any significant genome-wide or local genetic correlations. For the most part, the pattern of phenotypic associations was similar to that of the genetic correlations, with significance likely driven by power. Additionally, the pattern of local genetic correlations and crosstrait lead SNP concordance provide a more nuanced characterization of the association between cortical brain structure and the blood immune traits and generally reflect the pattern of genome-wide genetic correlations. These similarities suggest a genetic basis for the phenotypic associations.

Our enrichment analyses helped us identify potential mechanisms linking cortical brain structure and the blood immune markers. Surface area–immune genes were enriched for catabolic biological processes as well as astrocyte and neuronal genes. The well-defined roles of astrocytes in the blood-brain barrier, supplying nutrients from blood to neurons, providing neuroimmunity, and modulating synaptic activity (48), provide an opportunity to play a key role in the immune crosstalk between blood and brain (49). Overall, this implies that the link between surface area and immune markers is likely mediated by astrocytes. The enrichment results for thickness-immune genes provided less mechanistic insights. However, one overlapping gene, *DPYSL5*, which was implicated in interneuron and inhibitory neuron enrichment, encodes a protein involved in neuronal development (50).

Evidence of a genetic overlap between immune markers and brain structure may have further implications for brain disorders. We found that both surface area–immune and thickness-immune genes were at least nominally enriched for schizophrenia-associated genes. A recent study discovered that genes shared by cortical brain structures and schizophrenia were enriched for inflammatory and immune markers (51). Additionally, the nominal enrichment for Parkinson’s disease genes, among surface area–immune genes, included the gene *SLC30A10*. This gene is among the astrocyte-specific genes and is involved in maintaining manganese levels (52,53), which is a biological process that has been implicated in the pathophysiology of Parkinson’s disease (54,55). It is possible that some individuals are more susceptible to brain-related disorders given a greater genetic propensity for crosstalk between brain and blood.

Surface area exhibited greater associations with the blood immune markers than cortical thickness. This may be related to lifespan variation in cortical structure and immunity. Cortical thickness peaks earlier than surface area in childhood (56). This may allow more time for immune-related genes and surface area to interact during development. The other end of the lifespan is marked by neurodegeneration and immune dysregulation. Our results suggest that a subset of the population may have a greater propensity for reduced brain structure, with elevated blood immune markers and possibly exaggerated neurodegeneration and immune dysregulation in late life. Supporting this possibility is our finding that Parkinson’s disease–related genes were nominally enriched among the surface area–immune genes. Moreover, Parkinson’s disease is associated with immune dysregulation (57).

Monocytes had the largest genetic overlap with cortical thickness and surface area, which may suggest a link between cortical structure and innate immunity, presumably through inflammation. The first wave of monocytes have a similar embryonic origin as microglia, which are the brain’s resident immune cells (19). Therefore, a genetic propensity for increased monocytes peripherally may be mirrored by a larger population of microglia centrally. One possibility is that a genetic propensity for a larger proportion of monocytes and microglia may lead to stronger peripheral-central crosstalk during inflammatory states, with potentially negative implications for cortical structure. This is speculative, of course, and further investigation of this potential mechanism is warranted.

There are several limitations of this study. Most of the genetic tools that were utilized rely on European references; therefore, in the main analysis, we used GWAS summary statistics from individuals of European ancestry only. We did, however, use a GWAS from an East Asian sample for the validation of lead SNPs. Additionally, the gene mapping strategy employed for conjFDR loci used expression quantitative trait loci and chromatin interaction sources from brain and blood tissue, which may bias enrichment analyses, although the tissues selected were directly relevant to the phenotypes studied. The proportions of WBC types may also be related to cortical brain structure. We opted to use WBC counts due to their clinical utility. However, the use of cell proportions would likely have minimal impact on the current results due to a high genetic correlation with cell counts (58).

## Conclusions

Here, we presented phenotypic associations and the shared genetic architecture between cortical brain structure and 7 clinically relevant blood immune markers. We found that the genetic overlap between cortical structure and the immune markers exhibited mixed effect directions that probably represent the complex relationship between the brain and the immune system. The biological underpinnings implicate neural cell types that may mediate these associations. Moreover, we discovered a consistent enrichment for genes associated with schizophrenia, a disorder with neurodevelopmental origins that has been linked to alterations in cortical brain structure and the immune system. These findings inform potential mechanisms underlying the relationship between the brain and blood immune markers, with important implications for brain development and the etiology of brain-related disorders.

## Supplementary Material

SupplementaryTables

SupplementaryInformation

## Figures and Tables

**Table:** KEY RESOURCES TABLE

Resource Type	Specific Reagent or Resource	Source or Reference	Identifiers	Additional Information
Add additional rows as needed for each resource type	Include species and sex when applicable.	Include name of manufacturer, company, repository, individual, or research lab. Include PMID or DOI for references; use “this paper” if new.	Include catalog numbers, stock numbers, database IDs or accession numbers, and/or RRIDs. RRIDs are highly encouraged; search for RRIDs at https://scicrunch.org/resources.	Include any additional information or notes if necessary.
Antibody	NA			
Bacterial or Viral Strain	NA			
Biological Sample	NA			
Cell Line	NA			
Chemical Compound or Drug	NA			
Commercial Assay Or Kit	NA			
Deposited Data; Public Database	UK Biobank	https://doi.org/10.1038/s41586-018-0579-z	application no. 27412	
Genetic Reagent	NA			
Organism/Strain	NA			
Peptide, Recombinant Protein	NA			
Recombinant DNA	NA			
Sequence-Based Reagent	NA			
Software; Algorithm	LD score regression	https://doi.org/10.1038/ng.3211		
Software; Algorithm	LAVA	https://doi.org/10.1038/s41588-022-01017-y		
Software; Algorithm	MiXeR	https://doi.org/10.1038/s41467-019-10310-0		
Software; Algorithm	conjFDR	https://doi.org/10.1371/journal.pgen.1003455		
Software; Algorithm	FUMA	https://doi.org/10.1038/s41467-017-01261-5		
Transfected Construct	NA			
Other	NA			
