# Supplementary material for: Genetic Overlap Between Global Cortical Brain Structure, C-Reactive Protein, and White Blood Cell Counts
Source: Biol Psychiatry. Author manuscript; Available in PMC 2024 Dec 30. (PMC11684752; doi:10.1016/j.biopsych.2023.06.008)
Supplement: SupplementaryInformation [file NIHMS2041558-supplement-SupplementaryInformation.pdf]

# SUPPLEMENTARY INFORMATION

# Genetic Overlap Between Global Cortical Brain Structure, C-Reactive Protein, and White Blood Cell Counts

Parker *et al.*

## Supplementary Methods

### Phenotypic Data and Analysis

Measures of cortical thickness and surface area were acquired from the UK Biobank (application no. 27412). A total of 40,055 participants had measures of global cortical thickness and surface area. Details on the acquisition and pre-processing of MRI scans as well as the extraction of imaging derived phenotypes have been previously described (https://biobank.ctsu.ox.ac.uk/crystal/refer.cgi?id=1977). In brief, brain imaging was conducted across four sites using an approximately 35 minute protocol which includes 6 imaging modalities. Processing of 1mm isotropic T1-weighted images included defacing and assessment of quality using a semi-automated pipeline. Gradient distortion correction was performed and brain extraction was completed using MNI152 standard space template. FreeSurfer v6.0 was used to estimate cortical thickness and surface area. Global cortical thickness was estimated as the average thickness of the left and right hemisphere. While global surface area was estimated as the sum of left and right hemisphere surface area.

Several immune markers were also acquired from the UK Biobank and were used to assess the phenotypic association between cortical brain structure and blood immune markers. At the initial recruitment visit, all consenting participants provided blood samples that were used to assay WBC counts (basophil, eosinophil, lymphocyte, monocyte,

neutrophil, and leukocyte count) and levels of C-reactive protein. A total of 478,168 participants had these blood immune markers assayed. A smaller fraction of participants (n=5865) also provided blood samples at the time of their MRI scan (only at Cheadle MRI site). Details regarding the processing of blood samples for complete blood counts have previously been described (https://biobank.ndph.ox.ac.uk/ukb/refer.cgi?id=1453). In brief, Beckman Coulter LH750 instruments were used to measure white blood cell counts within 24 hours of blood collection. For quantification of CRP and other blood biochemical markers, a separate analysis of blood biochemistry was conducted for samples collected at baseline only (https://biobank.ctsu.ox.ac.uk/crystal/refer.cgi?id=5636).

Linear models were used to assess the association between cortical brain structure (surface area and thickness) and each blood immune marker (CRP and WBC counts). Prior to modelling, participants with a history of stroke, dementia, or psychiatric diagnoses were excluded. All blood immune markers were log transformed, with the exception of basophil and eosinophil count which were square root transformed. Next, all cortical and immune metrics were standardized to Z-scores. Outliers were removed based on values that exceeded four standard deviations from the mean. Finally, the linear models included age, $age^2$, sex, body mass index and MRI site as covariates. Additionally, for models using baseline values of blood immune markers, the difference in age from baseline to time of imaging was included as a covariate.

Sensitivity analyses were conducted including systolic blood pressure and lifetime smoking status (binary) as covariates (Model A). The sample size was reduced with the addition of these covariates. Therefore, to test the effect of reduced power, a separate model (Model B) was run using the same reduced sample but with the original model unadjusted for blood pressure and smoking.

## Sources of Genetic Data

To investigate genetic overlap between cortical brain structure and the blood immune markers, summary statistics from large-scale GWAS were acquired. For cortical surface area and thickness summary statistics were acquired from the ENIGMA Consortium based on the recent study of 33,992 participants of European ancestry (1). For the conjunctional FDR analyses (see below) we acquired summary statistics excluding the UK Biobank cohort to avoid sample overlap. For replication, a separate GWAS of n=33.735 UK Biobank participants was utilized (2). GWAS summary statistics for CRP was acquired from the Neale Lab based on the analysis of 361,194 UK Biobank participants of European ancestry (https://docs.google.com/spreadsheets/d/1kvPoupSzsSFBNSztMzl04xMoSC3Kcx3CrjVf4yBmESU/edit#gid=178908679). For replication, a GWAS of 204,402 participants of European ancestry that was conducted by the CHARGE consortium was utilized (3). Finally, the Blood Cell Consortium (http://www.mhi-humangenetics.org/en/resources/) provided summary statistics for all of the WBC counts based on a recent study of 563,085 participants of European ancestry (4). To avoid sample overlap for conjunctional FDR analyses (see below), we used summary statistics derived from the UK Biobank sample only. For replication, summary statistics from 151,807 east Asian participants from the cross ancestry analysis by the Blood Cell Consortium was utilized (5). Supplementary table 18 provides a complete list of GWAS summary statistics used in this study.

The original GWAS for white blood cells measured each subtype using a relative count which was the total WBC (leukocyte) count multiplied by the proportion for each cell type (4). The total WBC count was measured by impedance and the percentage of each WBC type was measured using flow cytometry gates. We used WBC counts in our analyses particularly due to the common use in clinical practice. The proportion WBC types has a strong genetic correlation with WBC counts (6) and therefore the use of proportions is unlikely to alter the results of the genetic overlap.

## Genetic Associations using LDSC, LAVA, MiXeR, and conjFDR

Pairwise genetic correlations at the global (i.e., genome wide) and local (i.e., within a region of the genome) levels were quantified using linkage disequilibrium score regression (LDSC) (7) and local analysis of co-variant annotation (LAVA) (8), respectively. LDSC is a widely used genetic tool for estimating genetic correlations between pairs of traits using summary GWAS statistics. LAVA is a novel tool designed to perform local genetic correlations, at a default of 2495 genomic loci. For each locus, we used the default threshold for heritability (p=0.05). To adjust for multiple comparisons, FDR correction was applied across all pairwise comparisons. Regional genetic correlations are better situated to capture genetic associations that exhibit mixed effect directions. That is, a pair of traits may exhibit no global genetic correlations as a result of an equal number of positive and negative (opposite effect directions) local genetic correlations of similar magnitude. Both LDSC and LAVA adjust for sample overlap, therefore, summary statistics from the original (full sample) GWAS of cortical brain structure and blood traits were used in these analyses.

To estimate genetic overlap between pairs of traits we used bivariate MiXeR v1.3 (https://github.com/precimed/mixer) which is a technique that is agnostic to effect directions (9,10). MiXeR uses a Gaussian causal mixture model to estimate the total number of “trait-influencing” variants. In this case, a trait-influencing variant is a common variant with a direct effect on the trait of interest excluding effects due to LD. Parameters are estimated with 20 iterations of the mixture models and the mean of each estimate is quantified along with the standard deviation over the 20 iterations. Given two traits of interest, MiXeR models the number of trait-influencing variants unique to each trait (non-overlapping) as well as the number of shared trait-influencing variants (overlapping). To assess model fit, MiXeR employs the difference in Akaike information criterion between the MiXeR model and an infinitesimal model with a positive value indicative of good model fit as well as visual inspection of log-likelihood plots - a flattened curve (i.e., a lack of an obvious minimum) or lack of consistency across the 20 iterations suggest poor model fit. MiXeR is robust to

sample overlap and therefore, summary statistics for the full set of samples in the original GWAS were used in these analyses.

To assess cross trait enrichment, conditional quantile-quantile plots (QQ-plots) are constructed. One trait is selected as the primary trait and QQ-plots are generated for several p-value thresholds (p=1, 0.1, 0.01, 0.001) in the secondary trait. A leftward deflection away from the null (diagonal) with a decrease in p-value threshold, is indicative of strong enrichment of a primary trait conditioned on the secondary. An observation of such cross trait enrichment for both traits conditioned on each other is a requirement for a valid conjunctional false discovery rate (conjFDR) analysis (https://github.com/precimed/pleiofdr) (11).

To identify genetic variants jointly associated with a pair of traits, we conducted a conjFDR analysis. This requires the estimation of the conditional false discovery rate (condFDR) values first. Enrichment observed in conditional QQ-plots are transformed to condFDR for each SNP. For a given p-value threshold, the FDR(p) is given by the proportion of null SNPs, $\pi_0$, multiplied by the null cumulative distribution function of all SNPs, $F_0(p)$, divided by F(p). As $\pi_0$ in GWASs of most phenotypes is close to one and F(p) can be estimated by the empirical cumulative distribution function, FDR(p) can be conservatively estimated by p/q. The conditional FDR is then defined as the posterior probability that a SNP is null for the primary phenotype given that the p-values for associations with both phenotypes are as small or smaller than their observed p-values. The condFDR statistic is calculated twice for a pair of traits such that each trait plays the role of primary and secondary trait. The conjFDR value is defined as the maximum of the two condFDR values for each SNP. The conjFDR is considered the posterior probability that a SNP is null for one or both traits given the p-values for associations with both traits are as small or smaller than their observed p-values. A significance threshold of conjFDR<0.05 was applied for all analyses. These analyses were conducted over 500 iterations of random pruning with the exclusion of the major histocompatibility region (MHC; chr6:25000000-34000000) and 8p23

inversion (chr8:7200000-12500000). All conjFDR analyses were conducted using non-overlapping GWAS samples.

## Gene Mapping and Quantification of Overlapping Loci

The functional mapping and annotation (FUMA) procedure (https://fuma.ctglab.nl/) was used to identify independent loci and map those loci to genes. Genetic variants identified by conjFDR were clumped using FUMA's default LD thresholds of $r^2 \geq 0.6$ for identifying independent significant SNPs and $r^2 \geq 0.1$ for identifying lead SNPs. To annotate conjFDR identified loci, positional, expression quantitative trait loci (eQTL), and chromatin interaction mapping were all considered. For position mapping the FUMA default window of 10kb distance from a gene was used. Databases from both brain and blood tissue were included for gene mapping using eQTLs. The eQTL databases included: GTEx v8 (for blood and brain), PsychENCODE, BRAINEAC, and eQTLGen. Additionally, genes mapped using chromatin interaction were included using the PsychENCODE and HiC databases. Ultimately, a conservative procedure was applied to obtain a final list of mapped genes. Only genes that were identified by at least two of the three mapping procedures (position, eQTL, chromatin interaction) were defined as mapped to a particular locus. For the main analysis, this gene mapping procedure was applied for loci shared by surface area (or thickness) and a combined list of all immune traits. Additional, analyses for each pairwise comparison also used the same gene mapping procedure.

Overlapping loci shared between cortical brain structure and each of the immune traits were identified using the "foverlaps" function in R package "data.table". This was carried out using the clumped genomic loci that were identified using FUMA.

To determine the pattern of effect directions across shared loci, lead SNPs shared between each cortical brain structure and immune trait pairing were selected. For each pairwise comparison, the proportion of lead SNPs with discordant effect directions in the original GWAS for the two traits was calculated. The results of these comparisons illustrate the degree of mixed effect directions. Values close to 100% represent a high degree of

discordance, values close to 0% represent a high degree of concordance, and values close to 50% represent a mixture of concordant and discordant effects.

### Lead SNP Validation in Independent Samples

Using an independent GWAS sample, replication of lead SNP sign was tested. An exact binomial test was conducted to determine if the proportion of lead SNPs with concordant signs in the original and replication sample was significantly above 50%. Lead SNPs for surface area and thickness were aggregated across the blood immune traits. The GWAS samples used in the original and replication analyses can be found in supplementary table 1.

## Enrichment Analyses

Enrichment analyses were conducted using (1) a combined list of genes mapped to a cortical structure (thickness or surface area) and all immune markers (CRP and WBCs), and genes mapped to cortical structure (thickness or surface area) and each individual immune marker (i.e., each pairwise association). FUMA GENE2FUNC was used to determine enrichment for gene ontology (GO) groups.

Cell specific gene sets were acquired from three human brain resources and one mouse brain resource. From fetal human cortex tissue, we used cell type genes from the single cell RNAseq study by Bhaduri et al (2021) (12). Additionally, we acquired PsychENCODE data on cell specific genes derived from bulk tissue in both fetal and adult human brain where cell specific genes were determined based on deconvolution with external single cell data (http://resource.psychencode.org/) (13). From a study by Darmanis et al (2015), we also acquired a subset of cell specific genes identified in adult human cortex (14). Finally, we used cell specific genes identified from the single cell RNA sequencing analyses of the mouse cortex and hippocampus by Zeisel et al (2015) (15). Mouse genes were converted to their human homologs and those without homologous genes were

excluded. Prior to enrichment analyses, all gene symbols (for cell specific genes and conjFDR mapped genes) were harmonized using the “limma” package in R and filtered to be among the list of approved gene symbols from the HUGO Gene Nomenclature Committee (HGNC) (all HGNC gene symbols were also harmonized using “limma”). To test for overrepresentation of cell specific genes among mapped genes, a hypergeometric test was applied, and p-values were corrected for multiple comparisons using the Benjamini-Hochberg method.

Curated lists of disorder related genes were acquired from DisGeNET using the R package “disgenet2r”. Genes associated with several psychiatric (bipolar, depression, schizophrenia), neurodegenerative (Alzheimer’s and Parkinson’s), neurodevelopmental (autism and attention deficit hyperactivity disorder), neurological (stroke and multiple sclerosis), and peripheral inflammatory (irritable bowel disorder, Crohn’s disease, and rheumatoid arthritis) disorders were included. Similar to the cell specific gene set analysis, all gene symbols were harmonized using limma and those not included in the list of all HGNC approved gene symbols were excluded. A hypergeometric test was conducted to assess overrepresentation of disorder genes among conjFDR mapped genes and Benjamini-Hochberg method was used to correct for multiple comparisons.

# Supplementary References

1. Grasby KL, Jahanshad N (2020): The genetic architecture of the human cerebral cortex. *Science* 17.
2. Meer D van der, Shadrin AA, O’Connell K, Bettella F, Djurovic S, Wolfers T, *et al.* (2022): Boosting Schizophrenia Genetics by Utilizing Genetic Overlap With Brain Morphology. *Biological Psychiatry* 92: 291–298.
3. Ligthart S, Vaez A, Võsa U, Stathopoulou MG, de Vries PS, Prins BP, *et al.* (2018): Genome Analyses of >200,000 Individuals Identify 58 Loci for Chronic Inflammation and Highlight Pathways that Link Inflammation and Complex Disorders. *The American Journal of Human Genetics* 103: 691–706.
4. Vuckovic D, Bao EL, Akbari P, Lareau CA, Mousas A, Jiang T, *et al.* (2020): The Polygenic and Monogenic Basis of Blood Traits and Diseases. *Cell* 182: 1214-1231.e11.
5. Chen M-H, Raffield LM, Mousas A, Sakaue S, Huffman JE, Moscati A, *et al.* (2020): Trans-ethnic and Ancestry-Specific Blood-Cell Genetics in 746,667 Individuals from 5 Global Populations. *Cell* 182: 1198-1213.e14.
6. Astle WJ, Elding H, Jiang T, Allen D, Ruklisa D, Mann AL, *et al.* (2016): The Allelic Landscape of Human Blood Cell Trait Variation and Links to Common Complex Disease. *Cell* 167: 1415-1429.e19.
7. Bulik-Sullivan BK, Loh P-R, Finucane HK, Ripke S, Yang J, Patterson N, *et al.* (2015): LD Score regression distinguishes confounding from polygenicity in genome-wide association studies. *Nat Genet* 47: 291–295.
8. Werme J, van der Sluis S, Posthuma D, de Leeuw CA (2022): An integrated framework for local genetic correlation analysis [no. 3]. *Nat Genet* 54: 274–282.
9. Frei O, Holland D, Smeland OB, Shadrin AA, Fan CC, Maeland S, *et al.* (2019): Bivariate causal mixture model quantifies polygenic overlap between complex traits beyond genetic correlation. *Nat Commun* 10: 2417.
10. Holland D, Frei O, Desikan R, Fan C-C, Shadrin AA, Smeland OB, *et al.* (2020): Beyond SNP heritability: Polygenicity and discoverability of phenotypes estimated with a univariate Gaussian mixture model ((X. Zhu, editor)). *PLoS Genet* 16: e1008612.
11. Smeland OB, Frei O, Shadrin A, O’Connell K, Fan C-C, Bahrami S, *et al.* (2020): Discovery of shared genomic loci using the conditional false discovery rate approach. *Hum Genet* 139: 85–94.
12. Bhaduri A, Sandoval-Espinosa C, Otero-Garcia M, Oh I, Yin R, Eze UC, *et al.* (2021): An atlas of cortical arealization identifies dynamic molecular signatures. *Nature* 598: 200–204.
13. Li M, Santpere G, Kawasawa YI, Evgrafov OV, Gulden FO, Pochareddy S, *et al.* (2018): Integrative functional genomic analysis of human brain development and neuropsychiatric risks. *Science* 362: eaat7615–eaat7615.
14. Darmanis S, Sloan SA, Zhang Y, Enge M, Caneda C, Shuer LM, *et al.* (2015): A survey of human brain transcriptome diversity at the single cell level. *Proceedings of the National Academy of Sciences of the United States of America* 112: 7285–90.
15. Zeisel A, M͡oz-Manchado AB, Codeluppi S, Lönnerberg P, Manno GL, Juréus A, *et al.* (2015): Cell types in the mouse cortex and hippocampus revealed by single-cell RNA-seq. *Science* 347: 1138–1142.

## Supplementary Figures

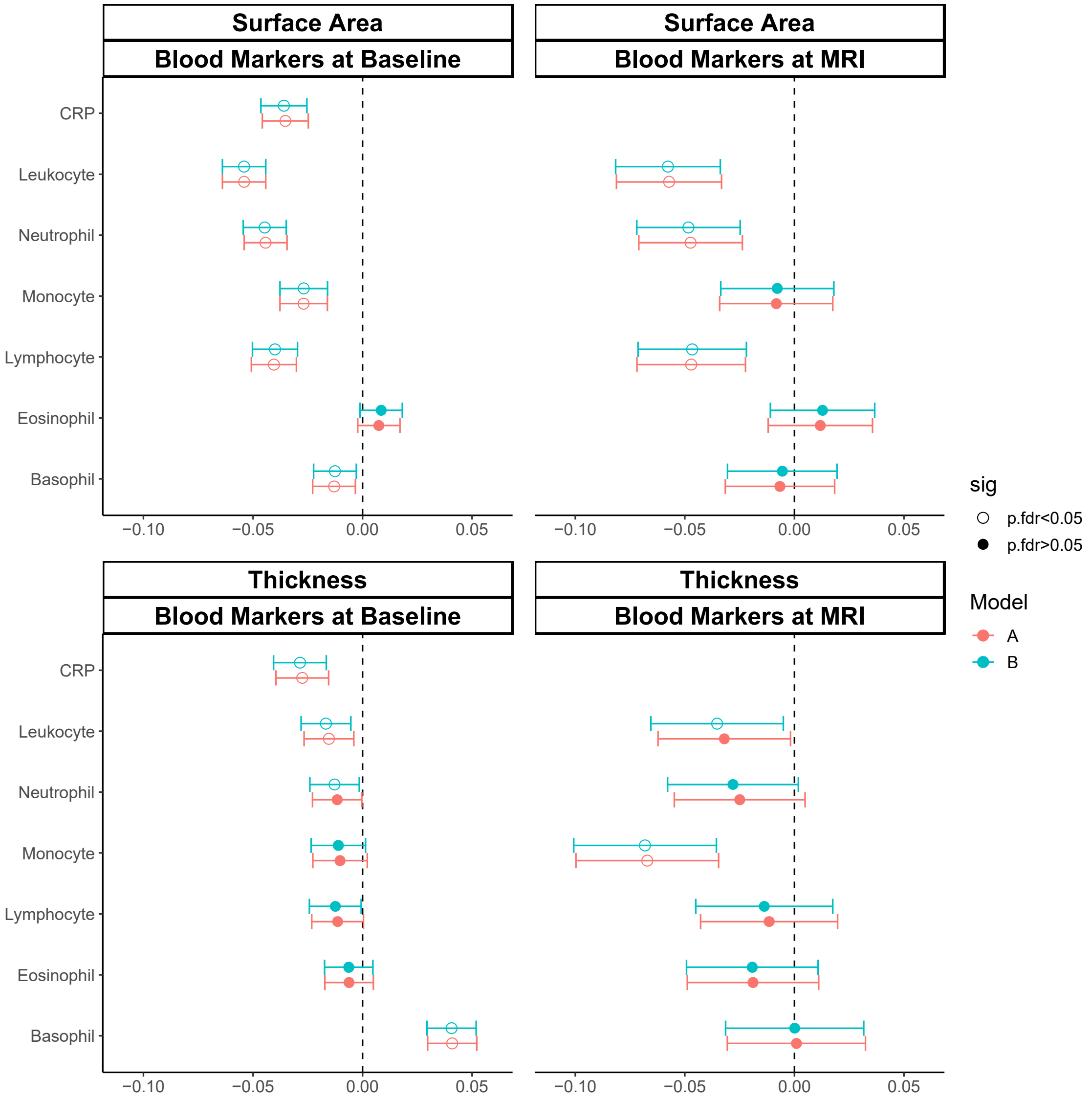


**Figure S1. Sensitivity Analyses of Phenotypic Associations.** The additions of systolic blood pressure and lifetime smoking status reduced the sample size to n=30,823 for blood markers at baseline and n=4966 for blood markers at MRI (Model A). This reduction in power reduced the number of significant associations, particularly for cortical thickness. Therefore, Model B (green) shows the results using the same sample as Model A without the addition of systolic blood pressure and smoking status (i.e., the model utilized in the main text). If there are differences in association between model A and B this suggests the additional covariates have an association with the cortical feature regardless of the reduction in statistical power. This was the case for thickness and neutrophil count measured with baseline blood samples as well as thickness and leukocyte count measured with blood samples taken at the time of MRI scan.

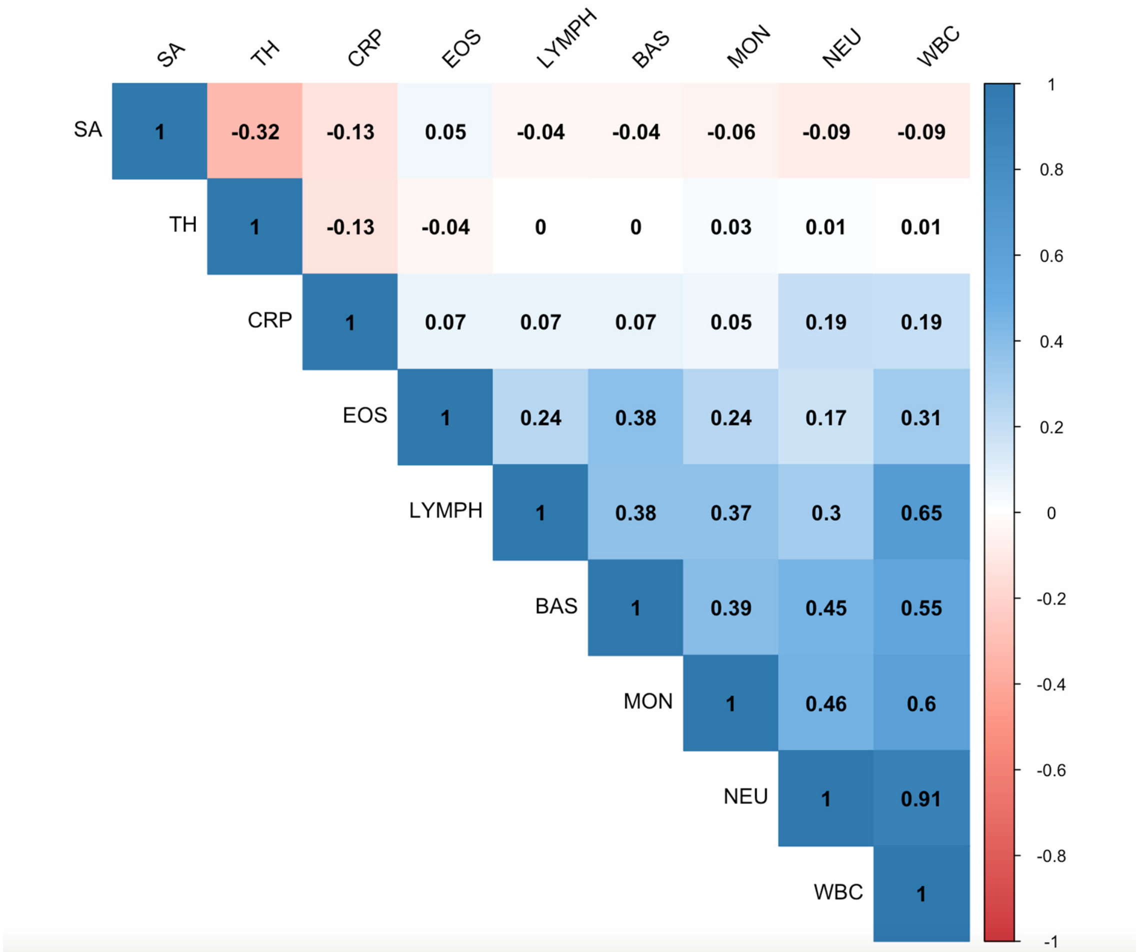


**Figure S2. Global Genetic Correlation Matrix.** LDSC global genetic correlation of all traits included in the analysis. SA: surface area; TH: thickness; BAS: basophils; EOS: Eosiniohils; LYMPH: Lymphocytes; MON: Monocytes; NEU: Neutrophils; WBC; Leukocyte/Total white blood cell count; CRP: C-reactive Protein.

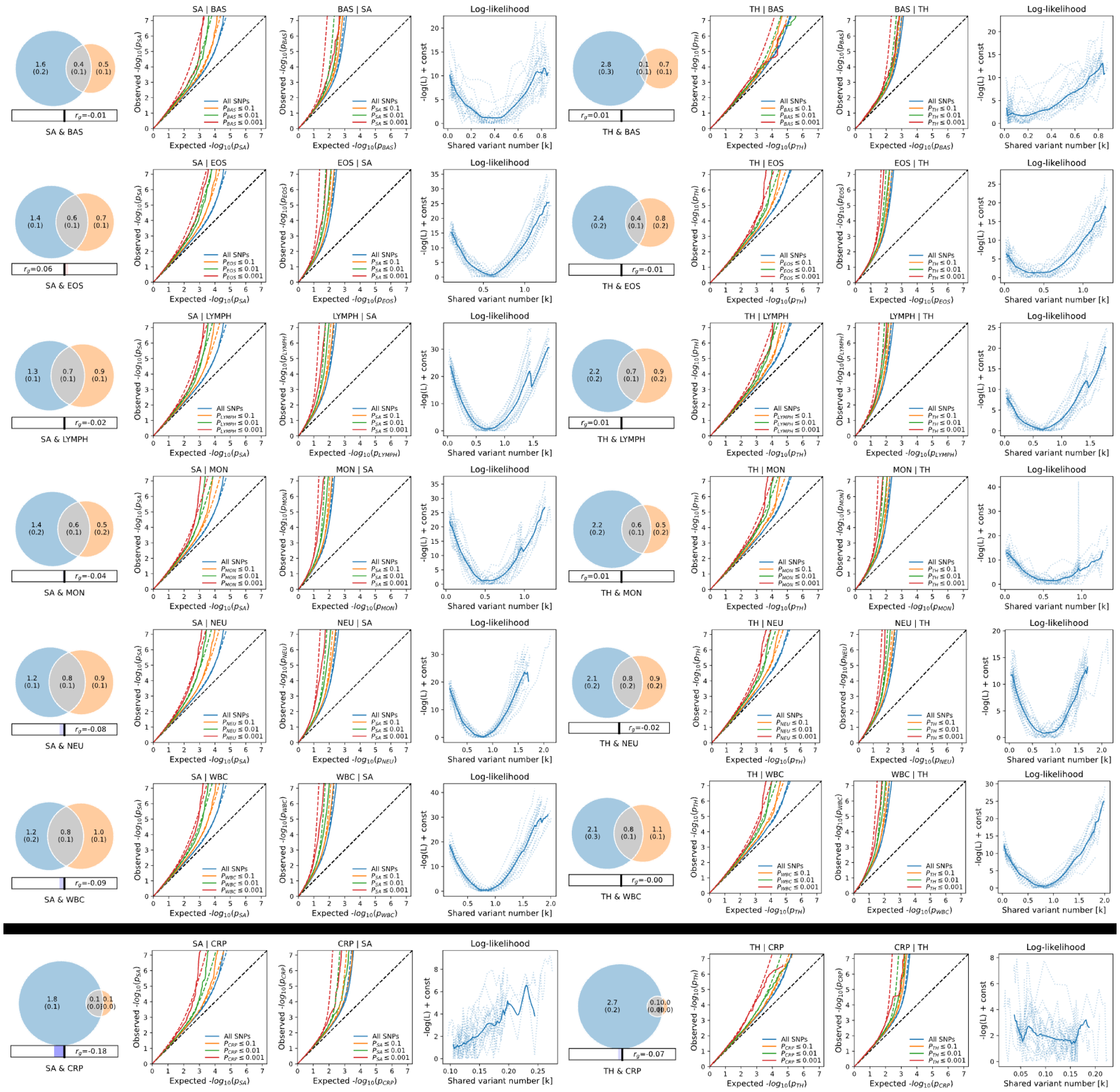

**Supplementary Figure S3. Bivariate MiXeR model output with log-likelihood plots.** Model Venn diagrams are displayed for each pairwise comparison are displayed. Beneath each Venn diagram is the MiXeR estimated genetic correlation for each comparison. Next, MiXeR derived qq-plots for conditional FDR for each pairwise comparison. Finally log-likelihood estimates for each of the 20 runs of MiXeR are displayed. Across the 20 runs there is a general lack of consistency for CRP analyses. Additionally, the log-likelihood plots for TH & CRP is generally flat a sign of poor model fit. SA: surface area; TH: thickness; BAS: basophils; EOS: Eosiniohils; LYMPH: Lymphocytes; MON: Monocytes; NEU: Neutrophils; WBC; Leukocyte/Total white blood cell count; CRP: C-reactive Protein.

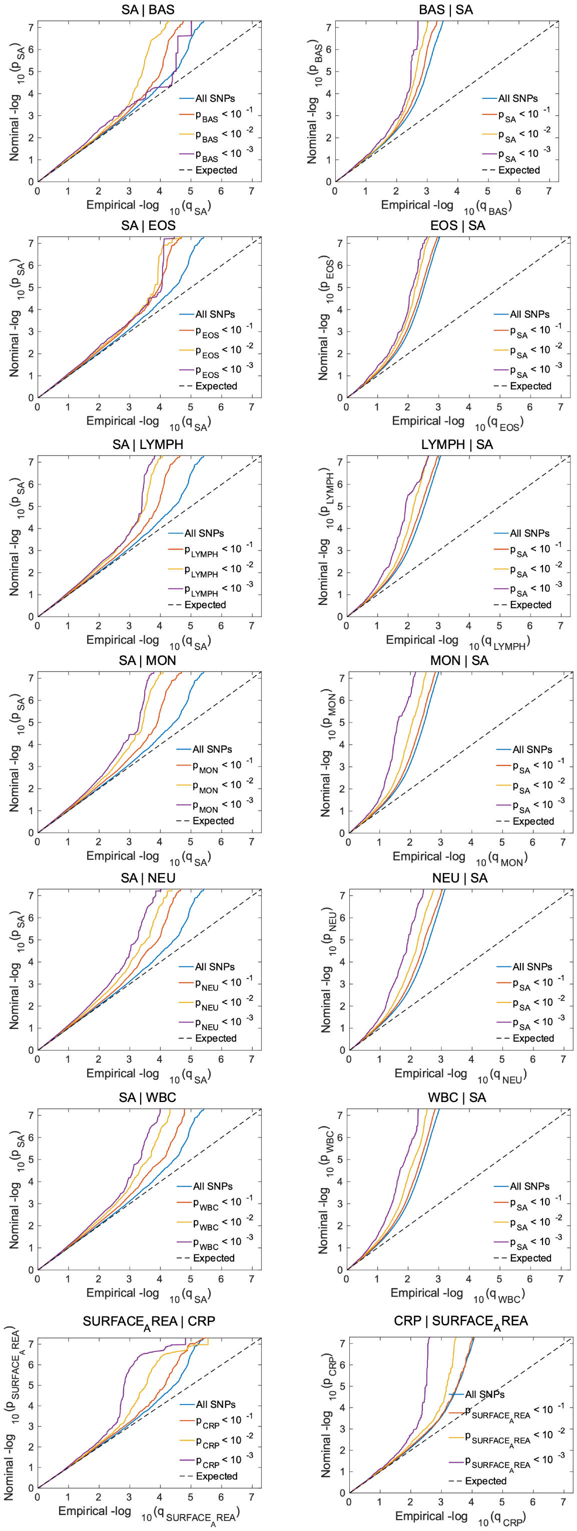


**Figure S4. Surface Area and Inflammatory Traits condFDR QQ-Plots.**
SA: surface area; BAS: basophils; EOS: Eosiniohils; LYMPH: Lymphocytes; MON: Monocytes; NEU: Neutrophils; WBC; Leukocyte/Total white blood cell count; CRP: C-reactive Protein.
